# Natural History of Irritable Bowel Syndrome in Women and Dysmenorrhea: A 10-Year Follow-Up Study

**DOI:** 10.1155/2012/534204

**Published:** 2012-03-14

**Authors:** Linda Bjork Olafsdottir, Hallgrimur Gudjonsson, Heidur Hrund Jonsdottir, Einar Björnsson, Bjarni Thjodleifsson

**Affiliations:** ^1^Department of Gastroenterology, Landspitali University Hospital, 101 Reykjavik, Iceland; ^2^The Social Science Research Institute, University of Iceland, 101 Reykjavik, Iceland; ^3^Faculty of Medicine, University of Iceland, 101 Reykjavik, Iceland

## Abstract

*Background*. Studies have shown that women are more likely to have irritable bowel syndrome (IBS) and more women seek healthcare because of IBS than men. *Aim*. We wanted to examine the natural history of IBS and dysmenorrhea in women over a 10-year period and to assess the change in IBS after menopause. *Method*. A population-based postal study. A questionnaire was mailed to the same age- and gender-stratified random sample of the Icelandic population aged 18–75 in 1996 and again in 2006. *Results*. 77% premenopausal women had dysmenorrhea in the year 1996 and 74% in 2006. 42% of women with dysmenorrhea had IBS according to Manning criteria in the year 2006 and 49% in 1996. 26% of women with dysmenorrhea had IBS according to Rome III 2006 and 11% in the year 1996. In 2006 30% women had severe or very severe dysmenorrhea pain severity. More women (27%) reported severe abdominal pain after menopause than before menopause 11%. Women without dysmenorrhea were twice more likely to remain asymptomatic than the women with dysmenorrhea. Women with dysmenorrhea were more likely to have stable symptoms and were twice more likely to have increased symptoms. *Conclusion*. Women with IBS are more likely to experience dysmenorrhea than women without IBS which seems to be a part of the symptomatology in most women with IBS. IBS symptom severity seems to increase after menopause.

## 1. Introduction

Irritable bowel syndrome (IBS) is a functional bowel disorder in which abdominal pain or discomfort is associated with defecation or a change in bowel habits and with features of disordered defecation [1]. Patients often experience additional symptoms such as bloating, sensation of incomplete evacuation, straining (constipation), and urgency (diarrhea) [2]. In the Western countries, more women than men seek health-care services for irritable bowel syndrome (IBS) [3, 4]. This can possibly be explained by factors involving physiological gender differences in gonadal hormones, stress reactivity, and inflammatory responses, as well as sociocultural differences in response to pain and/or bowel pattern changes [4]. In a recent study of men and women with IBS, the gender differences found were more complicated than a simple ration of men:women [5]. Women with IBS report more constipation, nausea, bloating, and extraintestinal and psychological symptoms than men with IBS [6]. Gender-related differences in gastrointestinal and somatic symptoms are apparent in persons with IBS but are most prominent in postmenopausal women [7]. Abdominal pain has been shown to be the most disruptive symptom in IBS and impacts on the quality of life in women with IBS [8]. The differences between genders in the occurrence of IBS could furthermore be the result of cultural, psychosocial, or healthcare access issues instead of purely physiologic differences [9].

Dysmenorrhea is a common conditions that can occur in 50–90% of women [10]. Dysmenorrhea is one of the most common complaints among women. It is defined as the presence of menstruation-associated spasmodic pain in the abdomen. Dysmenorrhea, dyspareunia, pelvic pain, and irritable bowel syndrome are common complaints among women of reproductive age and are not consistently associated with demographic risk factors [11]. Many women experience discomfort at the time of their periods. For most, this does not interfere with their daily lives or requires any special attention. However, for some women their monthly period is painful, problematic, and in some cases disabling. It can interfere with their lives because of the pain and inconvenience caused.

Dysmenorrhea severe enough to cause absence from work occurs in less than 5% of women [10]. Although improvement and worsening are equally likely for all women, improvement is more likely in women who bear children [10].

Population-based studies are essential for studying IBS since only a minority of IBS patients seek medical care [12]. Self-medication is common among these patients [12], and differences have been noted in IBS patients and nonpatients from the community [13, 14]. The great majority of IBS studies are patient or healthcare based. Women overall have a greater prevalence of IBS symptoms than men, particularly those associated with constipation [15]. Studies suggest that female sex hormones influence the severity of IBS symptoms [15]. A recent study suggests that an increase in gastrointestinal symptoms around the time of menses and early menopause occurs at times of declining or low ovarian hormones, suggesting that estrogen and progesterone withdrawal may contribute either directly or indirectly [16].

The objective of our study was to study the connection of IBS and dysmenorrhea in women and the change over a 10-year period. The secondary objective was to assess the change in IBS over menopause and the birth cohort effect of dysmenorrhea. The third objective was to assess the connection of functional gastrointestinal disorders (FGIDs) including functional dyspepsia (FD), heartburn and frequent abdominal pain (FAP), and dysmenorrhea severity.

## 2. Methods

### 2.1. Participants and Setting

In 1996 an epidemiological study of gastrointestinal diseases was performed in Iceland [17]. Involved were 2000 inhabitants in the age range of 18–75 years. The individuals were randomly selected from the National Registry. Equal distribution of sex and age was secured in each age group. In 2006 we attempted to contact all the individuals from 1996. A questionnaire was mailed to individuals at baseline, and the study questionnaire and an explanatory letter mailed to all eligible individuals. Reminder letters were mailed at 2, 4, and 7 weeks, using the Total Method of Dillman [18]. Individuals who indicated at any point that they did not want to participate in the study were not contacted further.

### 2.2. The Questionnaire

The bowel disease questionnaire (BDQ) [19, 20] was translated from English into Icelandic and modified for this study. The questionnaire was translated by two gastroenterologists and a pharmacist. A specialist in the Icelandic language at the University of Iceland made a linguistic modification. The questionnaire was piloted within a small group of IBS patients diagnosed by gastroenterologist.

The questionnaire was designed as a self-report instrument to measure symptoms experienced over the previous year and to collect the participant's past medical history [21].

The Icelandic version of the BDQ questionnaire addressed 47 gastrointestinal symptoms and 32 items that measure past illness, health care use, and socio-demographic and psychosomatic symptoms, together with a valid measure of non-GI somatic complaints, the somatic symptom checklist (SSC) [22]. There were only a few changes in the latter questionnaire (2006) which addressed 51 gastrointestinal symptoms and 33 items that measure past illness, health care use, and socio-demographic and psychosomatic symptoms. The questionnaire included questions on dysmenorrhea and the pain severity of dysmenorrhea.

### 2.3. Criteria to Identify Dysmenorrhea

Women were asked if they had menstruation. If they were menstruating, they were asked if they experienced dysmenorrhea in the beginning of their menstruation. Those who had dysmenorrhea were asked to state the magnitude of the dysmenorrhea pain; minor pain, medium pain, severe pain, very severe pain, and no pain. Menopause was identified in women who were menstruating in 1996 but reported that they did not menstruate in 2006.

### 2.4. Criteria to Identify IBS

The criteria for identification of IBS are presented in Table 1.

Diagnosis of IBS according to the Manning criteria [23] required two or more of the six symptoms listed in Table 1 and abdominal pain six or more times during the previous year [24, 25].

### 2.5. Transition between Disorders from the Initial to the Final Survey

**Figure 1:**
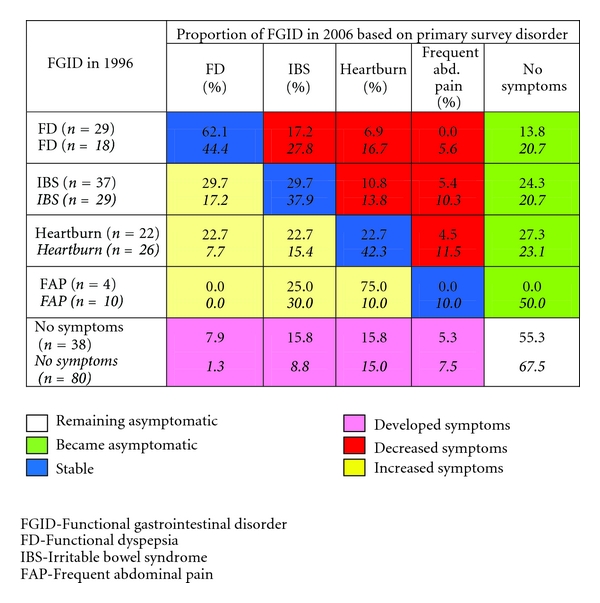
Transitions among symptom subgroups between the initial and final surveys. Women with dysmenorrhea, *women without dysmenorrhea. *

A transition model used by Halder et al. was modified and applied for this study [21]. The responses from the initial (1996) and final (2006) surveys were matched for each subject to examine the changes between disorders at an individual level for the 6 categories (IBS, FD, heartburn, frequent abdominal pain, and no symptoms). A 5 × 5 table was used to model these multiple changes and collapsed into 6 groups, as illustrated in Figure 1. Those with the most symptoms were prioritized higher. Those who developed more symptoms and those who reported fewer symptoms could be categorised into groups. There were six patterns of symptoms, identified as follows: (1) symptom stability, (2) symptom increase, (3) symptom decrease, (4) symptom onset, (5) becoming asymptomatic, and (6) none of these symptoms.

### 2.6. Mortality Data

For the 2006 survey, we identified all deceased individuals with the assistance of the National Registry of Iceland.

### 2.7. Statistical Analysis

Tables were constructed for frequency and percentage. Categorical variables were summarized using frequency and percentage. Chi square test was used to compare categorical variable. Type I error protection rate was set at 0.05. The exact *P* is listed in the tables and text. The statistical software SPSS (Statistical Package of Social Science) was used for data analysis.

### 2.8. Ethics

The National Bioethics Committee of Iceland and The Icelandic Data Protection Authority gave their permission for the research.

## 3. Results

### 3.1. Demographic Data of Involved Individuals

**Figure 2:**
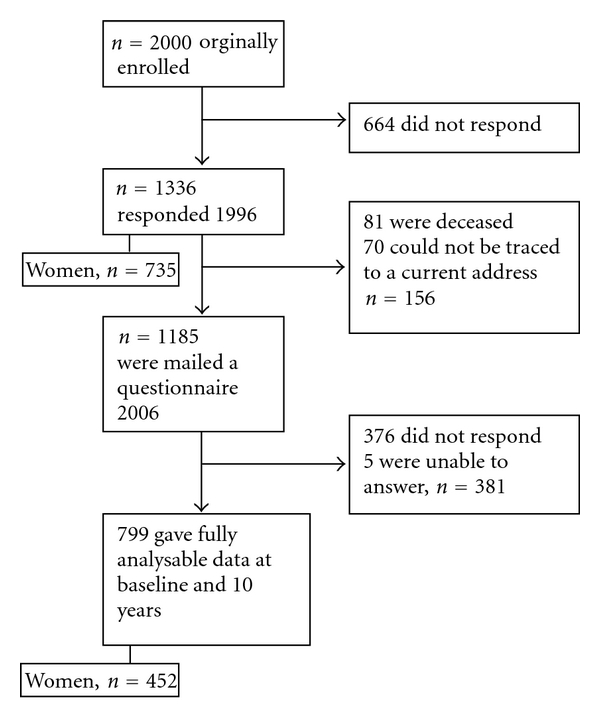
Flow of study participants.

In 1996 the response rate was 66.8% (1336/2000). Of the 1336 individuals who participated in 1996, 81 were deceased by 2006, five subjects were unable to answer, mainly because of old age, and 70 could not be traced to a current address. This left 1180 individuals, out of which 799 responded in 2006 (Figure 2). The response rate in 2006 was 63.7% (799/1255). The mean age of the individuals in 1996 was 43 and in 2006 53; there was not a significant difference between those who responded 2006 and those who did not respond (NS). Women were more apt to respond than men in both years. A larger proportion of women responded again in 2006 (57.8%) than those who responded in 1996 but did not respond again in 2006 (49.8%, *P* < 0.01). The responders represented the population concerning sex and age distribution. The response rate was higher for older subjects than younger ones. There was no significant difference between those who responded or those who did not respond in the year 2006, whether they were diagnosed with IBS in the year 1996 or not. Age distribution and demographic details of the study cohort are presented in Table 2.

### 3.2. Dysmenorrhea (Painful Menstruation)

**Figure 3:**
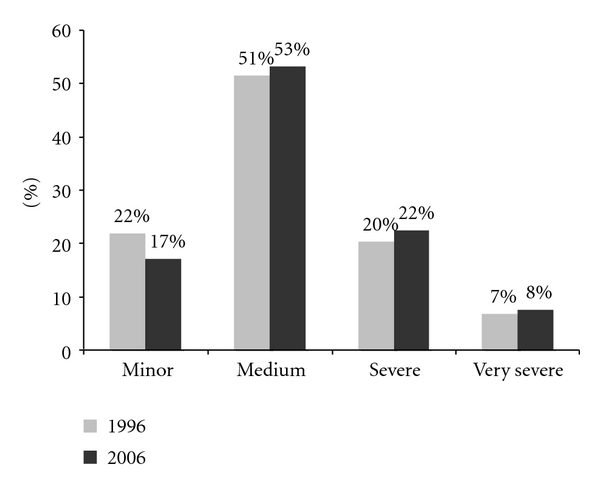
Distribution of dysmenorrhea severity (1996, *n* = 254, 2006, *n* = 152).

Of those women who responded, 331 women reported menstruation in 1996 and 205 in the year 2006. A total of 254/331 (77%) in 1996 and 152/205 (74%) in 2006 reported dysmenorrhea (Table 3). Half of those reported medium severity of dysmenorrhea. Slightly more reported minor dysmenorrhea in the year 1996 than 2006. Slightly more reported severe or very severe dysmenorrhea (Figure 3).

### 3.3. Dysmenorrhea and Irritable Bowel Syndrome

One out of ten women with dysmenorrhea had IBS according to the Rome III criteria in the year 1996, and 5% of women without dysmenorrhea had IBS (*P* = 0.170) (Table 4). A total of 39/152 (26%) women with dysmenorrhea had IBS according to Rome III in the year 2006 and 14/152 (9%) of women without dysmenorrhea had IBS, with a statistical difference (*P* = 0.013).

Overall 105/254 (41.5%) of women with dysmenorrhea had IBS according to the Manning criteria in the year 1996, and one out of four without dysmenorrhea had IBS, with a statistical difference (*P* = 0.014). 49% of the women in the year 2006 of women with dysmenorrhea had IBS according to Manning and 33% of women without dysmenorrhea had IBS in the year 2006 (*P* = 0.063).

### 3.4. Dysmenorrhea and Other Functional Gastrointestinal Disorders

**Figure 4:**
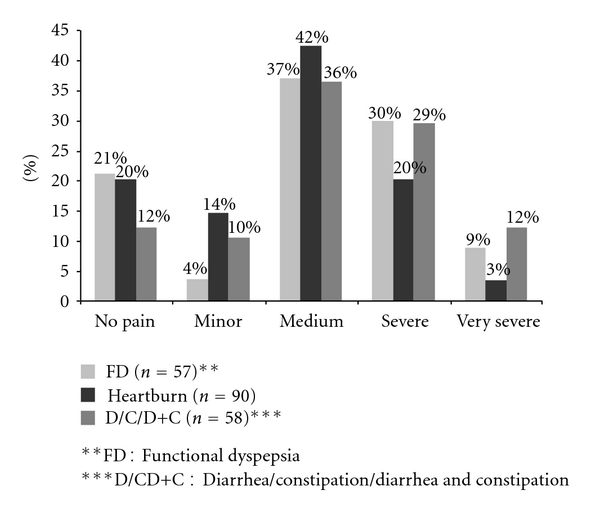
Functional gastrointestinal disorders and dysmenorrhea severity (2006, *n* = 152).

A total of 45/57 (79%) of those who had functional dyspepsia (FD) and 72/90 (80%) heartburn had dysmenorrheal, and 88% of those who had diarrhea and or constipation had dysmenorrhea (Figure 4). Altogether 39% of those who had FD and 41% of those who had diarrhea and/or constipation had severe or very severe dysmenorrhea. There was a significant statistical difference (*P* < 0.01) of dysmenorrhea between those who had FD and those who did not have FD. Those who had diarrhea and, or constipation had proportionally higher prevalence of dysmenorrhea than those who did not have diarrhea and or constipation (*P* < 0.01) (Figure 4).

### 3.5. Women with Dysmenorrhea in 1996 and after Menopause 2006, IBS, and Abdominal Pain Severity

**Figure 5:**
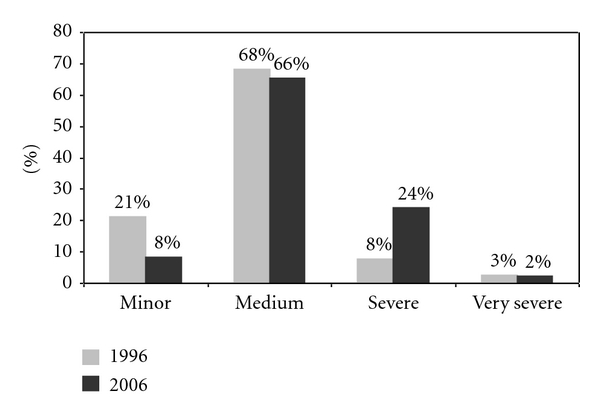
Abdominal pain in women with dysmenorrhea 1996 and after menopause 2006.

In the year 1996, overall 64 women experienced dysmenorrhea but did not have periods in the year 2006. In the year 1996, 24/64 (38%) of those had IBS according to the Manning criteria and 41% in the year 2006 altogether. 6% experienced IBS according to Rome III criteria in the year 1996 and 13% in the year 2006.  Figure 5 shows the changes in abdominal pain severity in women with dysmenorrhea in the year 1996 and after menopause in the year 2006.

### 3.6. Transition between Disorders from the 1996 and 2006 Surveys

As described in the method section, the groups in this analysis were defined as mutually exclusive, using the symptom hierarchy so that each subject appears in only one category for both the 1996 and 2006 surveys. There was a “no symptoms” category for those who did not meet any of the criteria applied for FGID. Due to the hierarchical classification, only a few participants occurred in some categories.

Transitions between disorders were explored in two ways, for women with dysmenorrhea and for women without dysmenorrhea (Figure 1). There was a substantial change in numbers in all the categories between the two surveys. The group “no symptoms” was the most common in both transition models. For the women with dysmenorrheal, the FD was the most stable one. A total of 17% moved into the IBS group and 14% into the no symptom group. IBS was stable in 30% cases, and the same number moved into the FD group. One-fourth moved into the no symptom group. There were only 4 women in the heartburn group of women with dysmenorrhea. In women without dysmenorrheal, the stability of symptoms was greater than that for those who suffered from dysmenorrhea. 44% of the FD group was stable between the initial and final surveys. One out of four moved into the IBS group. The stability for the IBS group was 38%. 5/29 (17%) moved into the IBS group and 21% into the no symptom group. The highest stability (42%) was in the heartburn group.

**Figure 6:**
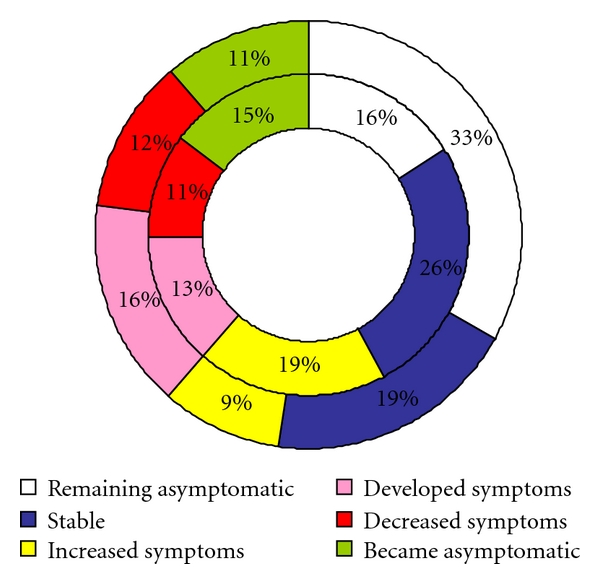
Six-group transition model, change from initial to the final survey. Women with dysmenorrhea (*n* = 130) in the inner circle and women without dysmenorrhea (*n* = 163) in the outer circle.

The transitions were collected into six groups. Comparison of the differences between women with and without dysmenorrhea in those transition groups (Figure 6) showed that the greatest difference was between the two groups of women who remained asymptomatic. The women without dysmenorrhea were twice more likely to remain asymptomatic than the women with dysmenorrhea. The women with dysmenorrhea were also more likely to have stable symptoms at followup than women without dysmenorrhea. The women with dysmenorrhea were twice more likely to have increased symptoms than women without symptoms.

### 3.7. Birth Cohort Effect of Dysmenorrhea

**Figure 7:**
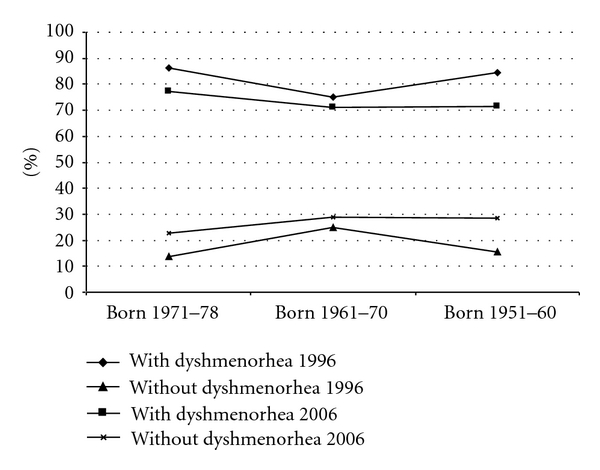
Birth cohort effect on the prevalence (%) in 10 years.

There was no significant difference in prevalence between birth cohorts in women with dysmenorrhea nor in women without dysmenorrheal (Figure 7).

## 4. Discussion

The current study makes it possible for the first time to follow up women with and without dysmenorrhea over a ten-year period and to observe how the FGID symptoms are associated with dysmenorrhea.

Every three out of four women with menstruation in this study experience dysmenorrhea which is similar to other studies. Analysis of women with dysmenorrhea showed that they were more likely to have IBS, either based on the Rome III criteria and/or the Manning criteria. The Manning criteria were much more sensitive than the Rome criteria. There was an increase in prevalence in dysmenorrhea over the ten-year period for both women with IBS according to Rome III and Manning, with more increase in dysmenorrhea in the Rome III group.

A meta-analysis based on a small number of studies compared gastrointestinal symptoms in pre- and postmenopause women [15]. The authors concluded that there was insufficient evidence to determine the effect of menopausal status on IBS symptoms. The current study demonstrated an increase in prevalence in women having IBS after menopause using both IBS criteria. Increase in gastrointestinal symptoms around the time of menses and early menopause occurs at times of declining or low ovarian hormones, suggesting that estrogen and progesterone withdrawal may contribute either directly or indirectly [16]. One study has shown that gastrointestinal symptoms burden was higher in postmenopausal women than in men, but these differences mostly disappeared when controlled for age [7].

Women with dysmenorrhea report more gastrointestinal symptoms prior to or concurrent with uterine cramping pain at menses than women who are non-dysmenorrheic [26]. Gastrointestinal symptoms tend to be increased across all cycle phases in women with IBS compared to healthy women, but both groups demonstrated a similar increase in severity immediately prior to or at the onset of menses [27]. The current study compared the FGIDs and dysmenorrheal severity and demonstrated that the great majority of women with dysmenorrhea had other FGID symptoms than those related to IBS. Women reported more severe abdominal pain after menopause than before. One study has shown that abdominal pain is the most disruptive IBS symptom [8].

The current study observed the transition between symptoms and revealed substantial difference between women with and without dysmenorrhea. Women without dysmenorrhea remained more often asymptomatic than women with dysmenorrhea. FGID symptoms were more stable in 10 years for women with dysmenorrheal, and they also had more increase in symptoms than women without dysmenorrhea. This demonstrated a difference between those two groups of women. The prevalence of menstrually related symptoms has been shown to be high and appears to affect bowel patterns [26]. A recent meta-analysis revealed a significant menstrual cycle effect for loose stools, bloating, abdominal pain, stool frequency, and other changes in bowel habit [15].

The strength of our study was the use of a stable and homogeneous population. The sample was randomly selected from the National Registry of Iceland and represented the nation as a whole in selected age groups. Only a minority of IBS patients seek medical care, and population-based studies are therefore essential for studying IBS. The population of Iceland with approximately 300 thousand inhabitants represents 1% of the whole population from all around the country.

There are some limitations to our study. The subjects were not specifically interviewed or examined to evaluate the possibility of organic disease. However, the 10-year (postal) followup went some way to making an organic cause of symptoms unlikely. Information on women using the contraceptive pill is lacking from this study, and this could affect the study. Also information on endometriosis and other gynaecological problems is not addressed in this study but could affect the results. Furthermore, since the response rate was 67% in 1996 and 69% in 2006, a drop-out bias cannot be excluded. A similar mean age in the respondent group and the nonrespondent group does not indicate an age dropout bias in the study.

## Figures and Tables

**Table 1:** Criteria to identify IBS.

Manning	
Pain eased after BM	
Looser stools at onset of pain	
More frequent BM at onset of pain	
Abdominal distension	
Mucus per rectum	
Feeling of incomplete emptying	

Rome III criteria	

Recurrent abdominal pain or discomfort at least 3 days/month	
in the last 3 months association with two or more of the following:	
Improvement with defecation	
Onset associated with a change in frequency of stool	
Onset associated with a change in form (appearance) of stool	

BMs, bowel movements.	
Subgroups of Rome III: subjects fulfilling the Rome III criteria	
were divided into 4 subgroups according to their bowel habits:	
(1) diarrhea-predominant (IBS-D), IBS-D is determined by	
predominantly loose or watery stools ≥25% of the time	
(2) constipation- predominant (IBS-C), IBS-C is determined	
by predominantly hard or lumpy stools ≥25% of the time	
(3) diarrhea and constipation (IBS-M), categories for mixed	
(mixed irritable bowel syndrome (IBS-M): meeting criteria	
for IBS-D and IBS-C ≥25% of time)	
(4) no diarrhea or constipation, un-subtyped (un-subtyped	
irritable bowel syndrome (IBS-U): not meeting criteria for	
of IBS-C nor IBS-D, that is, both are <25% of the time).	

*Rome III: *a close approximation of the Rome III criteria was used. The data were reevaluated retrospectively to conform to the Rome III criteria.

**Table 2:** Study population. Age and sex distribution.

	Population 2006 (%)	Respondents 2006 (%)
Gender		
Men	50.3	42.2
Women	49.7	57.8
Age		
28–35	19.5	14.52
36–45	24.9	20.40
46–55	22.8	22.15
56–65	15.6	19.52
66–75	10.4	15.14
76–85	6.8	8.26

Total number	*N* = 173859	*n* = 799

**Table 3:** Women with menstruation and dysmenorrhea.

	1996		2006	
Total women	446		444	
Women without menstruation	115	25.8%	239	53.8%
Women with menstruation	331	74.2%	205	46.2%
Women with dysmenorrhea	254	76.7%	152	74.1%

**Table 4:** Women with IBS according to Rome III and Manning and dysmenorrhea.

		1996	2006
Rome III	Dysmenorrhea	10.5%	25.7%
	Without dysmenorrhea	5.3%	9.4%
Manning	Dysmenorrhea	41.5%	48.6%
	Without dysmenorrhea	25.3%	33.3%
